# Associations between iKaluk/Arctic charr (*Salvelinus alpinus*) and estuarine benthic diatom habitats in nearshore Nunatsiavut waters

**DOI:** 10.1007/s00300-024-03323-z

**Published:** 2025-01-02

**Authors:** Zachary MacMillan-Kenny, Mary Denniston, Evan Edinger, Adam Templeton, David Côté, Audrey Limoges, Katleen Robert

**Affiliations:** 1 Department of Geography, Memorial University of Newfoundland and Labrador, St. John’s, NL Canada; 2 Fisheries and Marine Institute, Memorial University of Newfoundland and Labrador, St. John’s, NL Canada; 3 Department of Biology, Memorial University of Newfoundland and Labrador, St. John’s, NL Canada; 4 Northwest Atlantic Fisheries Centre, Fisheries and Oceans Canada (DFO), St. John’s, NL Canada; 5 Department of Environment, Nunatsiavut Government (NG), Nain, NL Canada; 6 Department of Earth Sciences, University of New Brunswick, Fredericton, NB Canada

**Keywords:** Arctic charr habitat, Video surveys, Indigenous knowledge, Acoustic telemetry, Benthic ecology, Arctic fish

## Abstract

iKaluk, Inuttitut for Arctic charr (*Salvelinus alpinus*), holds significant commercial and cultural value for Inuit communities throughout Nunatsiavut. Studies evaluating iKaluk habitat associations in freshwater are plentiful; however, there is limited information on the ecological makeup and sediment characteristics of anadromous charr habitats in marine environments. This study investigated the benthic associations of Arctic charr during their marine residency period in Nain, Nunatsiavut, using underwater videos, harvester-identified fishing locations, and acoustic telemetry. Drop-camera surveys were deployed on previously placed hydrophone acoustic receivers and within harvester-identified fishing locations to describe and quantify available benthic habitats in the study area. Telemetry information was used to identify charr occupancy hotspots, and calculate habitat suitability indices. A total of 248,056 benthic organisms belonging to 63 morphotaxa were identified within the 125 video drops used for benthic community characterization and these represented five faunal assemblages. Marine phase charr occupied estuaries to a greater degree than other fjord or coastal headland environments and these habitats were typically characterized by fine sediments covered by high densities of brittle stars (Ophiuroidea spp.) and benthic diatom mats. The consistent presence of diatomaceous sediments is indicative of abundant foraging opportunities. The importance of these habitats to iKaluk, need to be considered in future marine planning given the cultural and ecological value of this species to many northern communities that face growing threats from environmental change.

**Supplementary Information:**

The online version contains supplementary material available at 10.1007/s00300-024-03323-z.

## Introduction

In the context of widespread global concerns about climate change, Arctic and subarctic regions serve as critical indicators of the profound environmental transformations occurring. This is evident in the case of northern fish populations, where trophic dynamics are being disrupted, geographic ranges are in flux, and critical life history traits are being lost due to warming temperatures (Falardeau et al. 2017; Layton et al. 2021; Wight et al. 2023). Nunatsiavut, situated in Inuit Nunangat (Inuit homeland in Canada), stands at the forefront of these rapid environmental changes. Nunatsiavut is an Inuit self-governed area located in the northern part of Labrador, Canada, where heightened Arctic warming, and climate-driven environmental changes have caused shifts in the distribution of marine species and a loss of habitat (Ford et al. 2012; Cunsolo Willox et al. 2013). Since 1993, consistent annual warming has led to reduced ice coverage on both land and sea, accompanied by a notable alteration in fjord salinity in the region (Allard and Lemay 2012). These impacts are poised to intensify in the near future due to the current rate of climate change (Post et al. 2019), and important species for Inuit communities, like iKaluk—Arctic charr (*Salvelinus alpinus*) (Linnaeus 1758), are becoming increasingly vulnerable (Layton et al. 2021).

Arctic charr (*Salvelinus alpinus*), known as iKaluk in Inuttitut, exhibit high variability in habitat selection across their circumpolar distribution, which extends from the Arctic to northern temperate regions (Johnson 1980; Reist et al. 2013). Anadromous charr are integral to the economies, cultures, and food security of many northern communities (Friesen 2002; Day and Harris 2013; Roux et al. 2019; Dubos et al. 2023), including the Nain region of Nunatsiavut, Labrador, where the Arctic charr fishery is one of the most important marine resources (Kourantidou et al. 2022). The region’s charr population is divided into three stock complexes: Okak, Nain, and Voisey (Kourantidou et al. 2024). These stock complexes represent distinct geographic rearing areas and spawning rivers that are vital for the sustainability of local fisheries. The Nain stock complex, which is of particular focus here, includes important rearing rivers such as the Fraser River, Kamanatsuk Brook, and Kingurutik River in Nain Bay, as well as Ikadlavik Brook in southern Anaktalak Bay and other rivers found near Webb Bay (Côté et al. 2021). These rivers serve as key spawning and rearing habitats for charr before they transition to marine environments, where they forage and accumulate energy for their next migration back to freshwater.

In the sea, iKaluk inhabit a range of habitats stretching from protected estuaries to coastal headlands, but extensive use of estuarine environments is reported in the literature (Spares et al. 2015; Moore et al. 2016; Côté et al. 2021). Estuaries host a diverse range of prey, including benthic invertebrates and small fish that are integral to the Arctic charr diet, particularly capelin (*Mallotus villosus*) and sand lance (*Ammodytes* spp.), both recognized as key components of the charr diet in Nain (Dempson et al. 2002, 2008; Côté et al. 2021). Corroborating the preference for estuaries, community members and harvesters in Nain traditionally fish iKaluk within local estuaries during the spring and summer months. These fishing locations are vital for community members and have been passed down for generations for subsistence and economic gain.

Arctic charr typically migrate to the marine environment after smolting at around 7 years of age, spending the summer feeding before returning to freshwater for overwintering (Harris et al. 2020). Their marine phase is crucial for accumulating energy reserves, with the duration of ocean feeding migrations varying, often lasting around 30–45 days but sometimes as short as 6 days (Dutil 1986; Gyselman 1994; Harris et al. 2020). The timing and duration of estuarine residency during their marine phase can vary (Dempson and Kristofferson 1987; Klemetsen et al. 2003; Spares et al. 2015; Harris et al. 2020), but anadromous charr typically reside within and near estuarine systems. However, they may venture farther along the coastline to additional habitats (e.g., fjords, coastal headlands) while in transit to other estuarine systems (Moore et al. 2016) and/or for increased feeding opportunities elsewhere (Côté et al. 2021; Nordli et al. 2023). The influences motivating journeys beyond these estuarine systems have been linked to variations in sex, size, maturation, local environmental conditions, the availability of food resources, and the proximity to other river systems (Dempson and Kristofferson 1987; Spares et al. 2012, 2015; Côté et al. 2021).

The preferences and use of habitat by Arctic charr have been studied in freshwater environments (Sandlund et al. 2010; Sinnatamby et al. 2012; Murdoch and Power 2013), and numerous studies have examined their movements, including the timing of seaward migration and comparisons of occupancy between broader marine habitats such as estuaries, fjords, and coastal headlands (Spares et al. 2015; Moore et al. 2016; Harris et al. 2020; Côté et al. 2021). However, while these studies provide valuable insights into general movement patterns, detailed information on the specific habitat preferences and associations of charr in marine environments remains limited. This knowledge gap is critical because refuges like estuaries and other essential habitats for foraging are increasingly threatened by environmental changes, particularly in northern regions experiencing accelerated impacts of climate change (Bush and Lemmen 2019). Understanding these habitats can help predict and assess changes that may affect charr survival (Harris et al. 2020). A lack of comprehensive biological knowledge about such habitats limits our ability to fully understand the pathways in which environmental change affects iKaluk and hinders the sustainable management of species (e.g., Hutchings 1996; Walters and Maguire 1996; Foley et al. 2011).

The lack of ecological knowledge on iKaluk stocks, combined with coastal knowledge gaps and the threat of climate change in the region have sparked the development of the Imappivut—‘Our Oceans’ Marine Planning Initiative. This initiative was developed by the Nunatsiavut Government through consultation and collaboration with communities in order to manage and protect Labrador Inuit interests in the coastal and marine areas of Labrador. This study focuses on the marine iKaluk habitats in Nain, Labrador, Canada. Specifically, our objectives were to (1) investigate epifaunal-habitat associations using drop-camera surveys within acoustic receiver arrays and local harvester-identified charr fishing locations in Nain, Nunatsiavut; (2) explore benthic species-environment relationships within these habitats; and (3) assess benthic habitat availability and suitability for Arctic charr using habitat suitability indices.

## Methods

### Study area

**Fig. 1:**
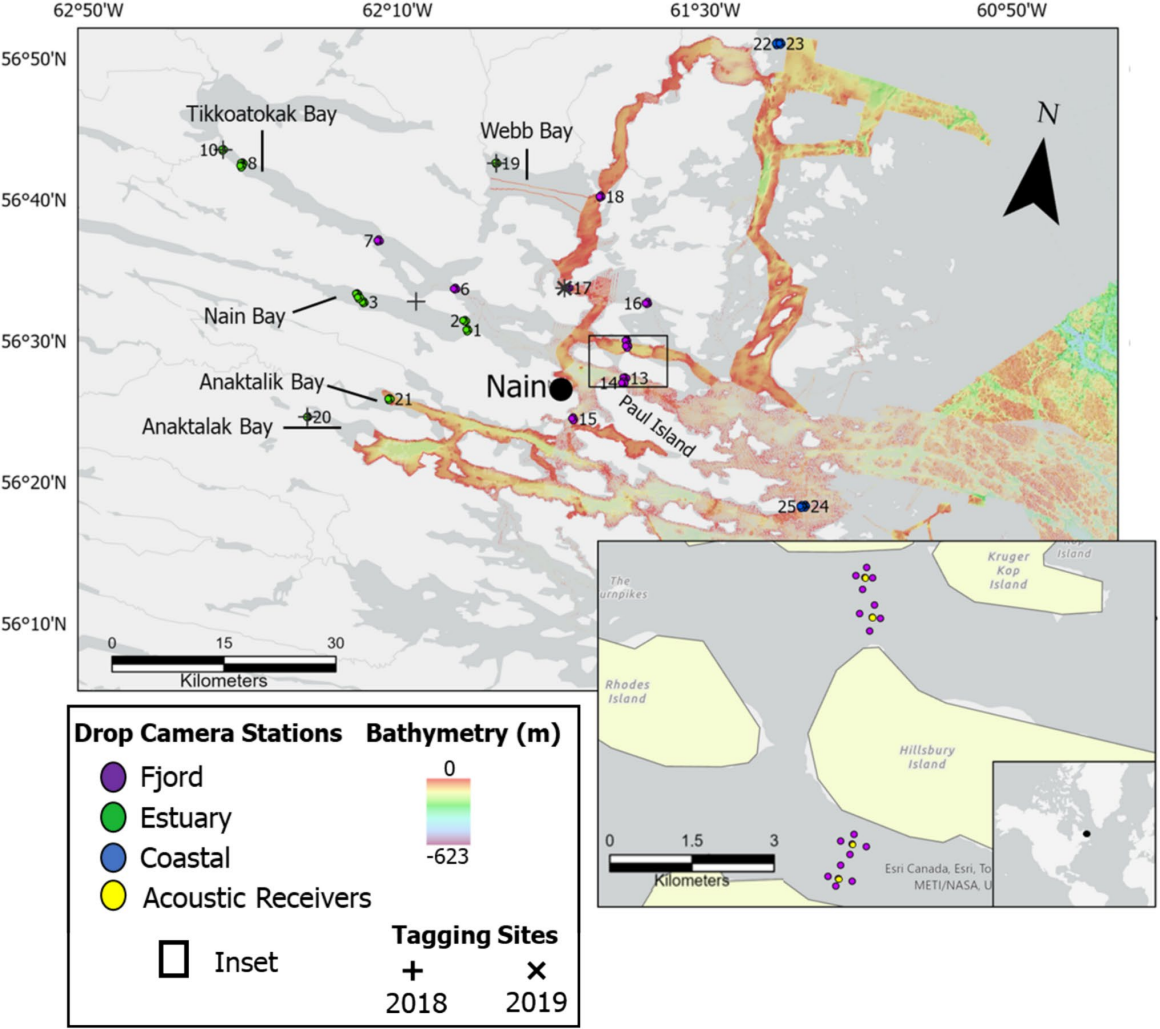
Drop-camera (*n* = 125) and acoustic receiver (*n* = 25) sites in the Nain region of Newfoundland and Labrador, Canada. Acoustic receivers are positioned in Nain Bay (*n* = 5), Tikkoatokak Bay (*n* = 5), Ocean Channel (*n* = 4), Anaktalak Bay (*n* = 1), Webb Bay (*n* = 4), and coastal headlands (*n* = 4). Acoustic receivers are located in the center of each cluster of 4 drop camera stations. Station numbers are listed near each station and coloured by habitat type. Canadian Hydrographic Service Non-Navigational (NONNA) bathymetric coverage with a spatial resolution of 100 m is shown in the background. The sites in Anaktalik Bay did not contain an acoustic receiver, but were community-identified as important fishing locations for Arctic charr

Our study is based in the Nain area of Nunatsiavut (56° N, 61° W; Fig. 1), a region at the transition between subarctic and polar climates. Its coast is open to the Labrador Sea and is characterized by rocky islets and deep fjords, forming an expansive archipelago teeming with marine biodiversity (Rangeley et al. 2022). Nain stands as the largest Inuit community in Nunatsiavut and holds the distinction of being one of the oldest permanent Inuit settlements in Canada. The marine environment is a cultural cornerstone and plays a crucial role for Inuit communities, providing subsistence and economic opportunities through species like iKaluk/Arctic charr. Despite the rich baseline ecological data derived by local knowledge holders throughout the community, Nain’s submerged marine habitats remain largely unmapped with western scientific tools. Consequently, there exists a limited understanding of the spatial distributions and roles of various habitats on its seafloor.

### Site selection

Assessments of Arctic charr habitats were made using a selection of sites that had existing information regarding their value as charr habitat. Specifically, surveys were placed surrounding the sites of acoustic receivers within a previously deployed telemetry array (Fig. 1). Generally, acoustic receivers (VR2W and VR2ARs, *Innovasea*) (*n* = 24) deployed by Fisheries and Oceans Canada (DFO) were positioned in river estuaries, fjords, and coastal headlands to record movements of tagged Arctic charr in the summer of 2018 and 2019, using a method designed to capture charr migratory pathways (i.e., anadromy) (Klemetsen et al. 2003; Côté et al. 2021). Many receiver locations overlapped with community-identified Arctic charr fishing areas, however, an additional identified fishing location in Anaktalik Bay that lacked an acoustic receiver was also included within this study (Fig. 1).

### Ground-truthing

Ground-truthing video surveys were used to classify benthic substrate and epifaunal communities at and adjacent to each study site (Fig. 1). These surveys were conducted using the DTPod drop-camera system by *Deeptrekker Inc*., which offers 1000-lumen LED lighting, two red-light scaling lasers (2.5 cm apart), and high-definition video (1920 × 1080, 30 fps) recording. Most hydrophone sites were surveyed between September 9 and 15, 2021, using the local vessel M/V *Safe Passage*, resulting in 95 video samples across 19 sites. In October 2022, additional surveys were conducted at coastal hydrophone sites and in Anaktalik and Anaktalak Bays using the M/V *Inuttatik*, producing 30 more videos. These surveys were particularly important for understanding habitats at the community-identified Anaktalik Bay site, which has long been known as an important Arctic charr fishing location. Each video survey spanned five minutes, covering an average distance of 59 m (Online Resource 1), ultimately resulting in 125 total videos collected across 24 acoustic receiver locations and one community fishing site. In addition, photographic transects on the R/V *William Kennedy* collected further benthic data at two coastal sites near Paul Island (sites 24 & 25), utilizing a custom drop-camera triggered by a hanging weight (Normandeau et al. 2018; Campbell & Normandeau 2019; MacMillan-Kenny 2024).

### Video analysis

Using the Monterey Bay Aquarium Research Institute’s (MBARI) Video Annotation and Reference System (VARS) (Schlining and Stout 2006), videos were annotated based on a randomized order to reduce human annotation biases (Durden et al. 2016). Every organism greater than 2 cm was counted and identified to the lowest taxonomic level (MacMillan-Kenny 2024). Taxa identification was performed with aid from experts and published species identification guides (Nozères and Archambault 2014; Nozères et al. 2014; Salvo et al. 2018; Lacasse et al. 2020).

**Fig. 2:**
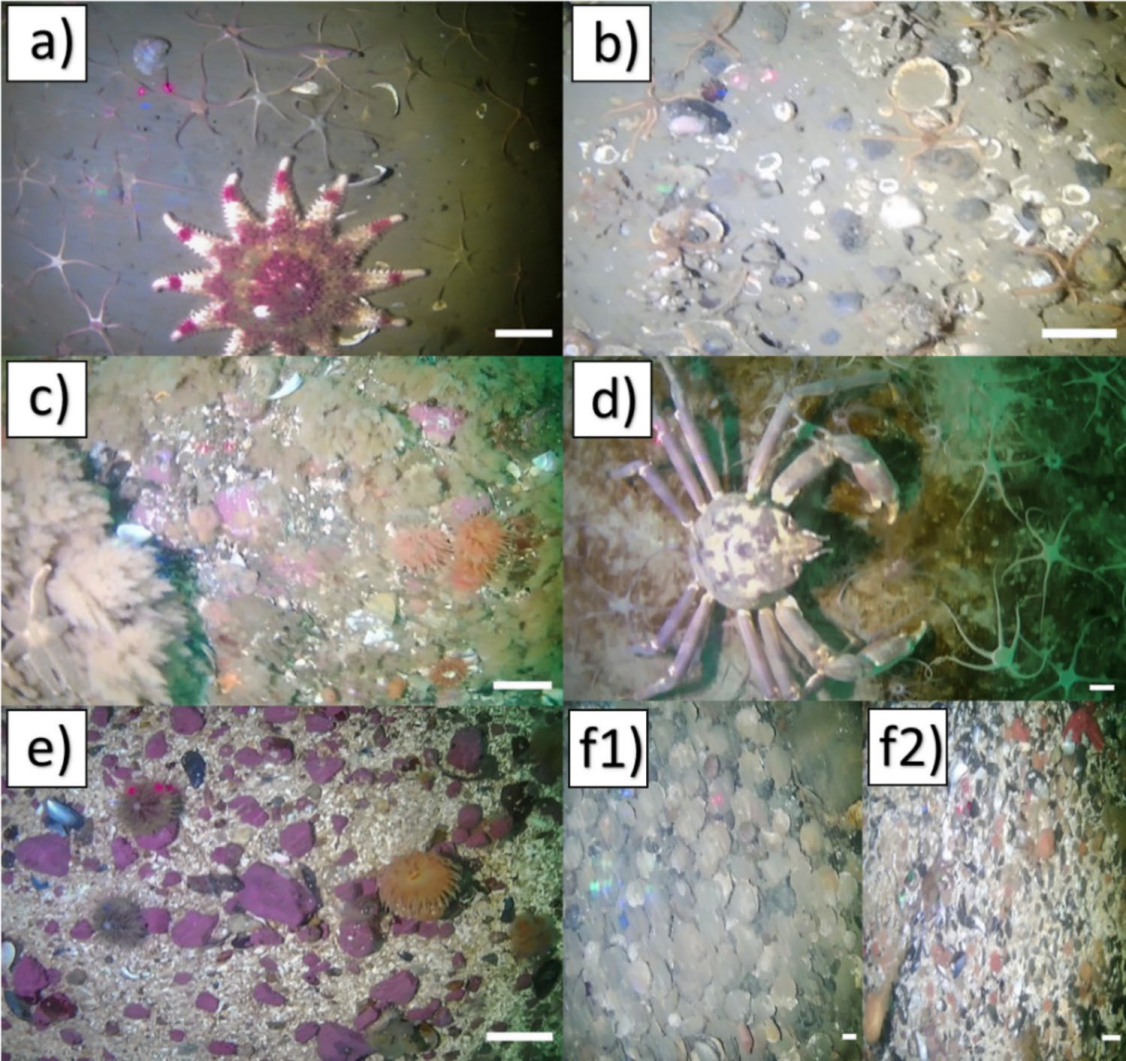
Images of the different substrate classes identified during video annotation: **a** fine sediments, **b** fine sediments with pebbles, **c** gravel mix with algal turf, **d** diatomaceous sediment, **e** gravel, **f1** scallop shell hash, and **f2** mussel shell hash. White bar for scale is 2.5 cm

All substrates (Fig. 2) observed in the survey were identified and classified with guidance from the Coastal and Marine Ecological Classification Standard (Federal Geographic Data Committee 2012). The substrate classes documented in this survey included: (A) fine sediments; (B) fine sediments with pebbles; (C) gravel mix with algal turf; (D) diatomaceous sediment; and (E) gravel. Scallop and mussel shell hash were only observed in small patches (F1, F2) (Fig. 2) and not included in the habitat suitability analysis (MacMillan-Kenny 2024). Using the software *Blender*, 1 frame for every 10 s intervals was extracted from each video and overlayed with 30 randomly placed points in *ImageJ*. Each of the randomly placed points were then assigned to a substrate category and the percentage cover of sediment in each image was calculated to determine substrate proportions per site (Online Resource 1). The distance between the two reference lasers was used to estimate sediment grain size.

### Epibenthic community analyses

All epibenthos abundance data obtained from the 125 video surveys were aggregated per hydrophone site. Taxa with three or fewer observations in all 25 sites were removed from the data set prior to analysis to reduce the variability caused by these low abundances (Brown et al. 2012a, b). The visual footprint (area ${\text{m}}^{2}$) of each image extracted for the substrate classification was computed using *Biigle’s* laser point detection tool (Schoening et al. 2015; Langenkämper et al. 2017). The summed visual footprint was used to estimate the approximate area (m^2^) of each transect and the total number of organisms per morphotype per transect was converted into densities. Using Bray–Curtis (1957) similarities, the distance for the species matrix was calculated. The species matrix was Hellinger transformed to reduce the effect of higher abundances (e.g., Ophiuroidea spp.) (Legendre and Gallagher 2001).

Cluster analyses (i.e., Hierarchical Clustering Analysis) were performed in *R* using the hclust function in the ‘cluster’ package. These clustering techniques were used to identify discontinuities and groupings of organisms within the data (Legendre and Legendre 2012; Brown et al. 2012a, b; Van Der Reijden et al. 2021). An average hierarchical clustering method (‘Unweighted Pair-Group Method using arithmetic Averages’—UPGMA) was selected to derive faunal assemblages from the Hellinger-transformed species density data (Sokal and Michener 1958; Borcard et al. 2018). To determine the size and cutoff of each cluster, fusion level values, silhouette widths and matrix correlations were optimized (Borcard et al. 2018). Analysis of similarity (ANOSIM), is a non-parametric statistical test, and was used to identify significant differences between and within clusters. To investigate the number of observed species relative to the sampling effort, species accumulation curves were derived (Gotelli and Colwell 2001). Species accumulation curves were created for each cluster.

To describe the biological composition of each assemblage the dominant taxa were derived. Dominant taxa consist of the most abundant species within a community that may influence the occurrence and distribution of other species and overall community structure (Smee 2010; Nemani et al. 2022). These organisms have abundances that are greater than the individual group assemblage mean (Borcard et al. 2018). Additionally, an Indicator Value approach was used to determine indicator taxa of each assemblage (Dufrêne and Legendre 1997). These values were derived using the indval function from the ‘labdsv’ package in *R* and are used to measure the association between a species and assemblage by assessing the relative abundance and frequency of each species per grouping. The function aims to identify an ideal indicator species found exclusively for each assemblage (Mouillot et al. 2002; Dufrêne and Legendre 1997). Because the indicator species are determined based on their specificity to one grouping, they are useful for monitoring changes to the sites associated with those specific groupings (McGeoch and Chown 1998; Kubosova et al. 2010).

### Modelling of benthic species-environment relationships

Prior to modelling the influence of environmental variables (Online Resource 1) on assemblages, a data exploration protocol was performed using methods from Zuur et al. (2010) (see MacMillan-Kenny 2024 for full methodology). In addition to the substrate percentage cover described previously, from the five video drops at each hydrophone, depth was measured using the vessel’s depth sounder and the mean value was calculated. Each drop location was measured to the nearest river mouth on ArcGIS Pro using the Geodesic measurement tool which is calculated in a 3D spherical space as the distance across the curved surface of the world (Online Resource 1). A mean distance to freshwater variable was then calculated per acoustic receiver site.

Using the Hellinger-transformed species matrix, a redundancy analysis (RDA) was performed using the Vegan package in *R* to summarize the variation in faunal density and to explain this variation using a set of explanatory variables. RDA is a canonical ordination approach that can be used to statistically test the relationships between environmental variables and species data (Legendre and Legendre 2012). To select the explanatory variables tested in the RDA, linear dependencies were explored by computing the *X* variables’ variance inflation factors (VIF) and a forward variable selection procedure was performed (Borcard et al. 2018). The number of explanatory variables were reduced to achieve model parsimony and avoid strong correlations among the explanatory variables. Only taxa with a goodness-of-fit of at least 0.65 in the ordination plane formed by axes 1 and 2 were represented in the RDA. To test the significance of the RDA, along with the explanatory variables and canonical axes, permutation tests were performed (*n* = 999).

### Telemetry

The DFO acoustic receiver data on Arctic charr from August 2018 to September 2019 (see Côté et al. (2021) for collection methodology) provided detection information from 18 receivers and 47 unique animal IDs (Online Resource 2). The charr were tagged and released during two field seasons in July/August of 2018 and 2019, and their movements were monitored from May to September each year, until they returned to their rivers for overwintering. The fish were caught using barbless single-hook fishing lines or gillnets with a 4½-inch (11.5 cm) mesh size. Each fish had a uniquely coded Innovasea V13 (36 × 13 mm, 9.2 g in air, tag life: 602 days) or V13T (46 × 13 mm, 9.7 g in water, tag life: 498 days) transmitter surgically implanted in their abdominal cavities. These transmitters emitted pulses at random intervals between one and two minutes, allowing the acoustic receivers to detect their presence. Prior to release, tagged Arctic charr were allowed to recover in a plastic tub equipped with air pumps and flowing seawater, ensuring minimal stress before their return to the environment (see Côté et al. 2021).

Of the 24 deployed acoustic receivers, two were lost (one in Nain Bay and one in Webb Bay) and four coastal receivers did not detect any tagged fish during their deployment. Habitats were previously classified as estuary (in close proximity to freshwater input), fjord (deep, glacially carved inlets), and coastal (headlands outside of fjords) (Côté et al. 2021). Of the remaining 22 acoustic receivers, 9 were located in estuaries, 9 in fjords, and 4 along coastal headlands (see Online Resource 2 for details) with an average depth of 48 m. To ensure accuracy, the detection data were imported into R and processed using the GLATOS package, which helped identify and remove potential false detections following the guidelines outlined by Holbrook et al. (2019). False detections occur when two or more transmitter signals collide, creating a false tag ID (Simpfendorfer et al. 2015). We used the ‘false_detections’ function in GLATOS, with a time threshold set at 45 min between consecutive detections, to filter out erroneous signals (Binder et al. 2017). Additionally, individual animals that were not detected at least twice by the same receiver at different times, or were not detected by more than one receiver, were excluded from further analysis (Binder et al. 2017; Murray 2022).

To examine residency time, the ‘detection_events’ function in GLATOS was used to distill 20,332 raw detection records into 87 distinct detection events. Detection events were defined as periods of sequential detections at the same receiver or groups of nearby receivers (depending on location) that were separated by a user-defined threshold period (Binder et al. 2017). This method standardizes detections, and accounts for duplicate detections where an individual could be detected at two nearby receivers during the same event. The threshold time used to distinguish events was set to 5 days (432,000 s), balancing the preservation of limited data with the need to avoid overestimating fish detection density between nearby locations. Fish residency time at each receiver was analyzed to create a rasterized movement map, highlighting local hotspots, defined as areas with high concentrations of Arctic charr moving through individual grid cells.

Fish movement was classified as either resident, where there are no movements between receivers, but are repeatedly detected at one location, or vagrant, where individuals were detected by at least two or more receivers (Béguer‐Pon et al. 2015; Murray 2022). A two-sample t-test was then used to determine whether the total length and weights of resident charr were significantly different from vagrant charr, as larger charr are more likely to travel farther from their natal river mouths (Dempson and Kristofferson 1987; Nordli et al. 2023). Movement between receivers was calculated using the ‘shortestPath’ function from the gDistance package in R, which calculates the least-cost distance between points in the water while using a land polygon as a boundary. The total estimated distance travelled, and the minimum distance dispersed from the release site was calculated for each individual fish and their movements were compiled to represent their pathways throughout the study sites in a heat map (see Hamoutene et al. 2018 for full methodology). The generated summary grid was smoothed for visual clarity by averaging each cell value with those in the surrounding two adjacent cells. Specifically, this involved considering values from a 5 × 5 grid centered on the point of interest, achieved through the ‘focal’ function in the Raster package. The density calculations were determined by the frequency of overlap between an estimated track and each grid cell (Hamoutene et al. 2018).

Habitat availability and suitability were calculated using a habitat suitability index (HSI) method outlined in Rudolfsen et al. (2021). The obtained value (0–1) estimates the suitability of habitat types for populations based on the species observed presence or absence within a dataset (Murray 2022; Rudolfsen et al. 2021). HSI was calculated based on the number of individual detections that occurred on the dominant substrate at each acoustic receiver location (Online Resource 3). For a substrate to be considered highly suitable, it must be used more than expected by chance, meaning its usage exceeds its proportional availability in the environment. If a substrate is used in proportion to its availability, it indicates no active selection by the species, whereas a higher-than-expected usage suggests a selection for that substrate. An average HSI was determined for each substrate class throughout the duration of the telemetry data (2018–2019). A chi-square goodness of fit test was used to determine if there was a significant difference in habitat use (observed) and available habitat (expected), based on the prediction that charr use all available habitats. The mean proportions of availability and use of habitat, which are metrics within the HSI calculation (see Rudolfsen et al. 2021), were used to estimate the number of charr that were expected and observed to use a particular habitat, respectively.

## Results

### Epibenthic community analyses

**Fig. 3:**
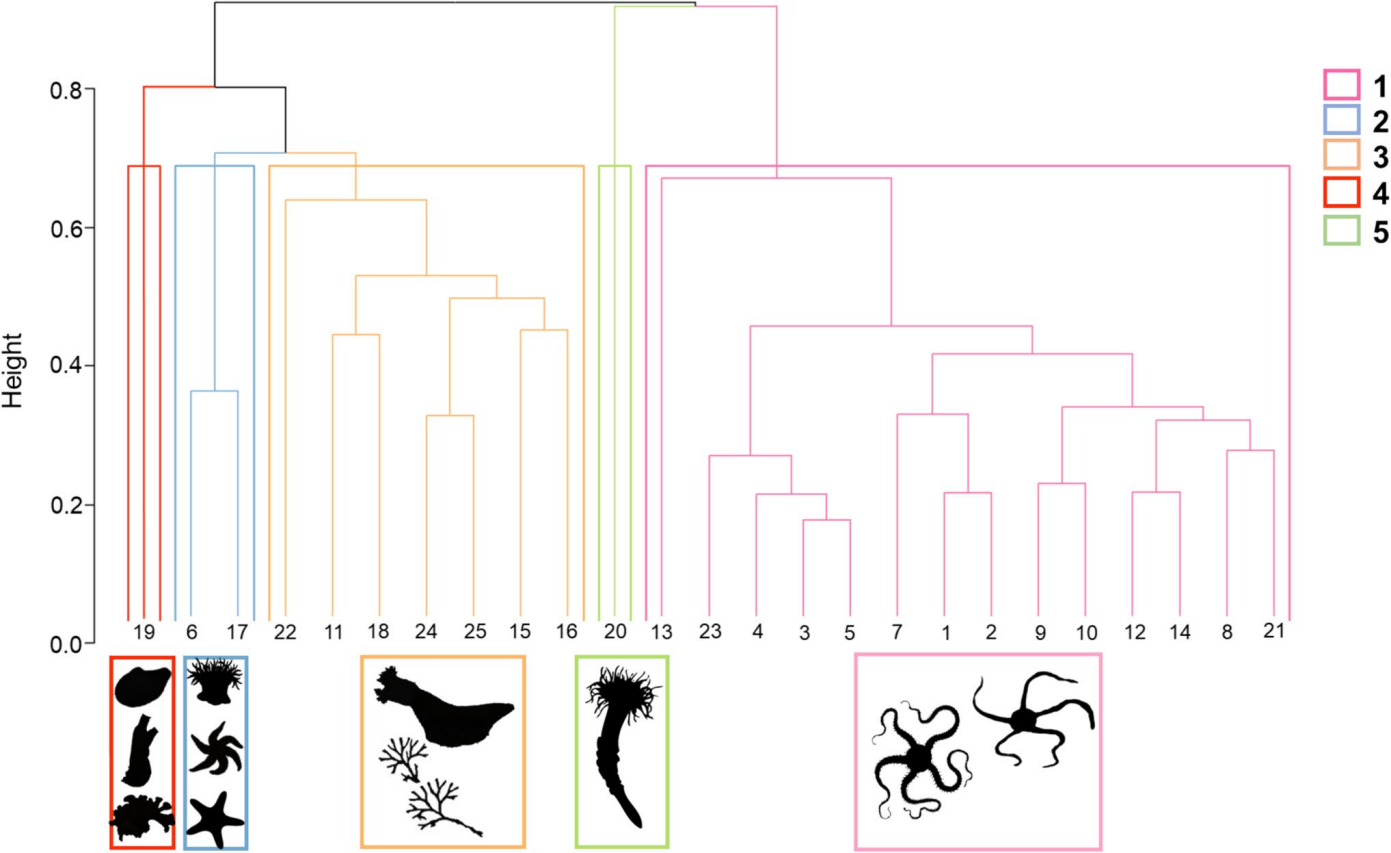
Unique assemblages of epifauna found in surveyed habitats based on hierarchical clustering (UPGMA). Indicator taxa silhouettes represent each assemblage

**Fig. 4:**
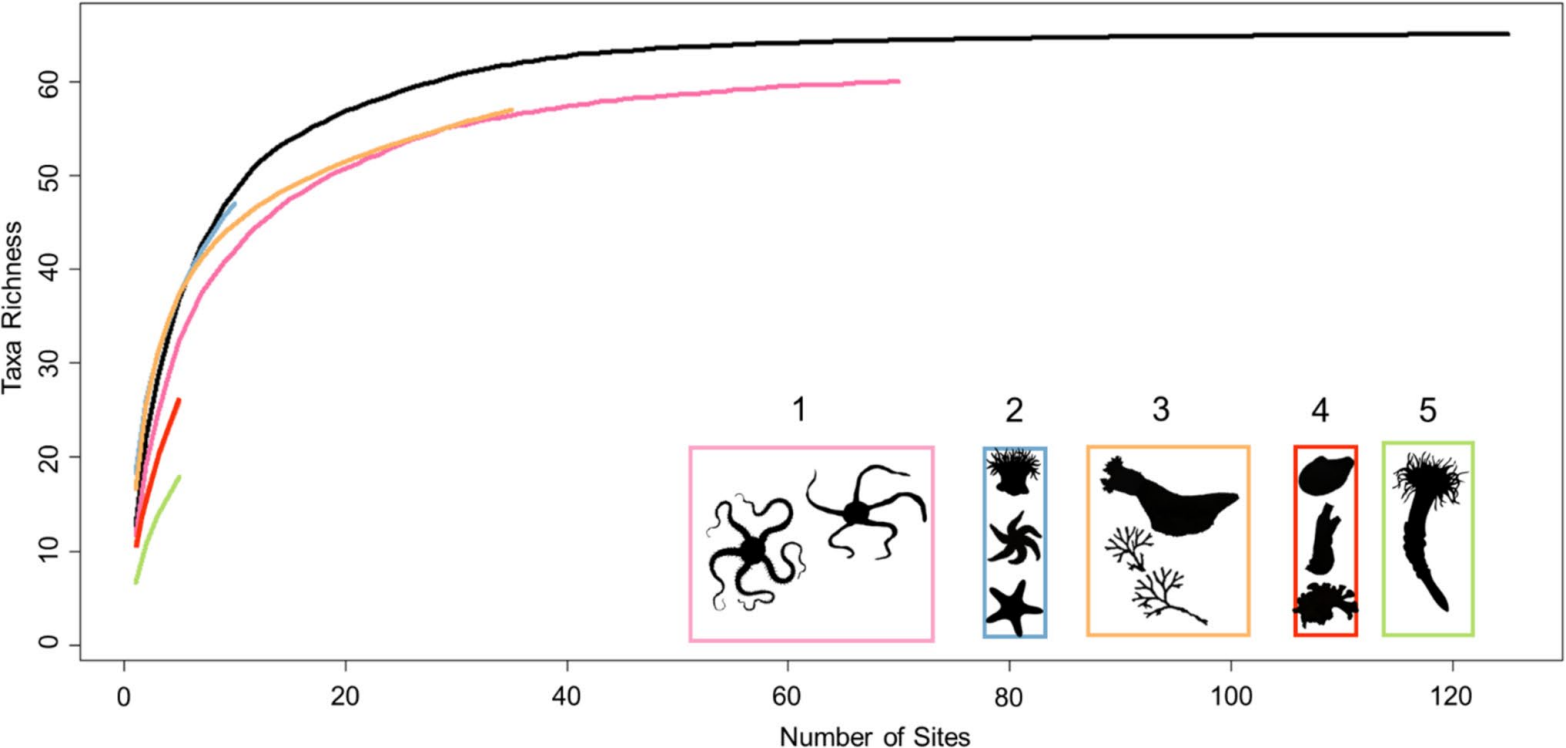
Species accumulation curves for each epifaunal assemblage derived from clustering. Includes the summation of species observed across all sites included in analysis (in black). *Y*-axis represents the number of morphotaxa observed; *X*-axis are the total number of sites sampled

**Fig. 5:**
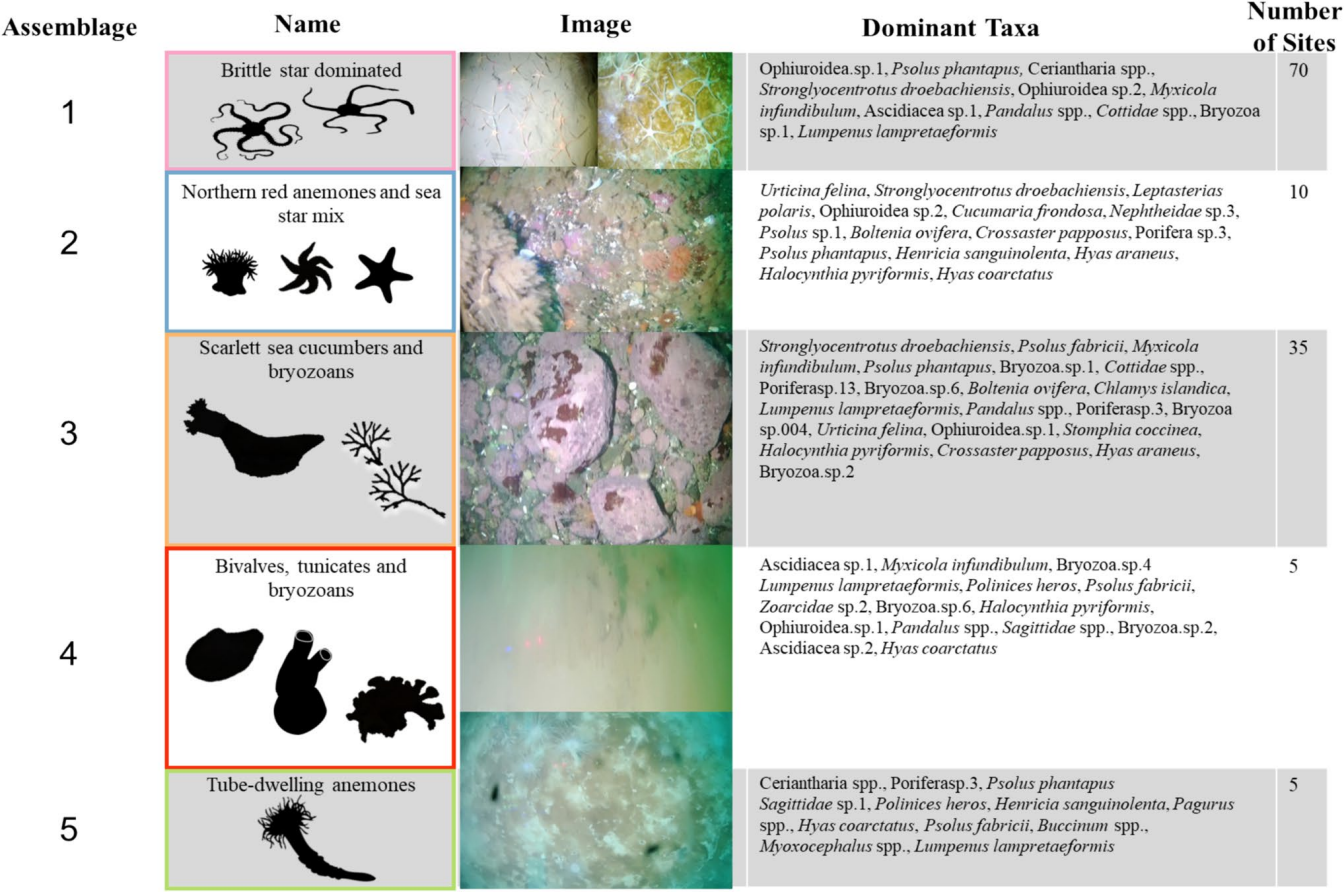
Images of epifaunal assemblages and dominant taxa observed for each species assemblages around Nain. Number of sites refers to the number of drop-camera stations in the survey

A total of 248,056 organisms belonging to 63 morphotaxa were identified within the 125 video drops used for community analyses and these were clustered into five faunal assemblages (Fig. 3). The ANOSIM statistic (*R* = 0.976) indicated a significant difference in species composition between the clusters (*p* < 0.001), suggesting distinct taxonomic profiles. Species accumulation curves for each assemblage revealed that assemblages 2, 4 and 5 were not sufficiently sampled because of their limited spatial coverage (Fig. 4). Assemblage 1 had the largest spatial extent, representing 56% of all video drops and occurring mainly in estuaries with fine sediments, diatom mats and evidence of bioturbation. This assemblage contained 223,700 individuals across 60 morphotaxa; however, the most commonly observed organism, and also this assemblage’s indicator taxa (Online Resource 4), was the brittle star (Ophiuroidea spp.) (*n* = 194,889) which created extensive agglomerations. The assemblage also contained 11 dominant taxa (Fig. 5) that included two potential local prey species for Arctic charr (sculpin, *Cottidae* spp. and snake blennies, *Lumpenus lampretaeformis*). Assemblage 2 represented 8% of all video drops and occurred within fjords dominated by a mixed gravel substrate sparsely populated by large boulders, pebbles, and algal turf. This assemblage was characterized by epibenthic fauna that attached to the underlying hard substrate, like northern red anemones (*Urticina felina*) (*n* = 5278) and sea stars (*Leptasterias polaris* & Asteroidea spp.). In addition to those indicator taxa, the assemblage contained 13 total dominant taxa. This assemblage contained 10,758 individual organisms across 48 taxa. Assemblage 3 had the second largest spatial extent, representing 28% of all video drops, occurring only in fjord and coastal habitats with fine sediments, pebbles, cobbles, boulders, and patches of algal turf. Characterized by scarlet sea cucumbers (*Psolus fabricii*) and bryozoans (Bryozoa spp.) as the indicator taxa, this assemblage held 27 dominant morphotaxa and 10,055 individual organisms across 57 taxa. Assemblages 4 and 5 were represented by only one site each. Assemblage 4 was located in an estuarine habitat and was comprised fine sediments that were mostly barren, but had small patches of epibenthic fauna. This assemblage contained 690 individual organisms across 26 taxa and was characterized by bivalves (*Portlandia arctica*), ascidians (Ascidiacea spp.) and bryozoans (Bryozoa spp.) as the indicator species. There were 16 dominant taxa within this assemblage, and the majority of these had low occurrences. Located near the estuarine habitat in Anaktalak Bay, Assemblage 5 was comprised fine sediments with a diatomaceous cover and was characterized by tube-dwelling anemones (*Ceriantharia* spp*.*) as the indicator taxon. This assemblage contained 2853 individuals across 18 taxa and showed similarities to Assemblage 1; however, it mostly lacked the presence of Ophiuroids (*n* = 1). There were 12 dominant taxa in this assemblage which included patches of sponges (Demospongiae spp.) and potential local prey species for Arctic charr (sculpin & snake blennies). These prey items were observed to be dominant taxa in all other assemblages as well.

**Fig. 6:**
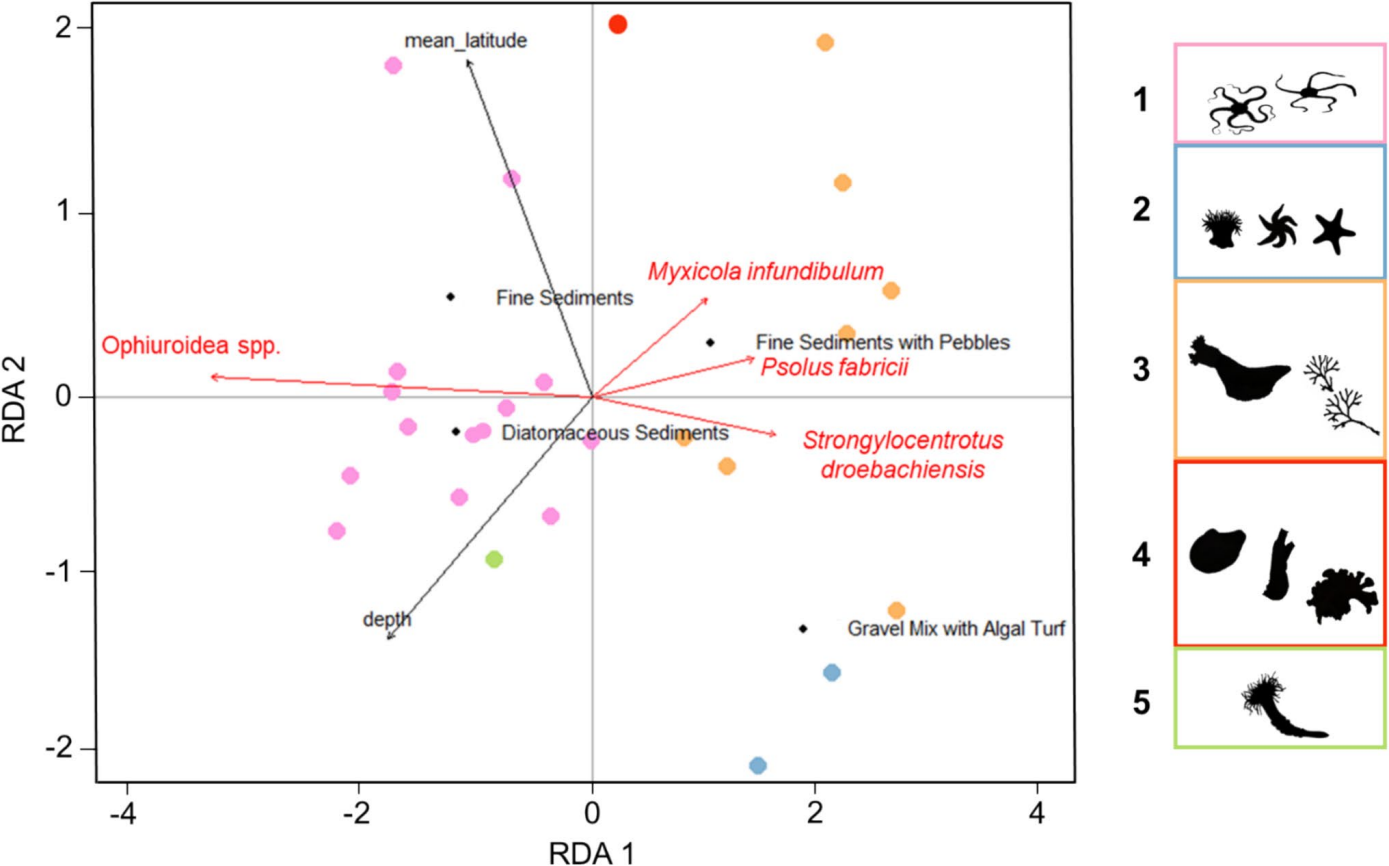
Taxa (< 0.65 goodness of fit) and environmental variable associations with assemblage types, based on parsimonious RDA. Points are coloured according to assemblage. Scaling shows the strength and effect of explanatory variables. Substrate classes include fine sediments, fine sediments with pebbles, diatomaceous sediments, gravel mix with algal turf

A redundancy analysis (RDA) was performed to investigate species-environment relationships. Six explanatory variables were retained after forward selection: depth, mean latitude, and the four substrate classes. The selected environmental variables significantly explain 44% (*R*^2^adj) (*p* = 0.001) of the variation in benthic taxa abundances and the most parsimonious model yielded two significant axes (RDA1: *p* = 0.001, *df* = 1, *F* = 18.230, RDA2: *p* = 0.039, *df* = 1, *F* = 3.243) (Fig. 6). The proportions of accumulated constrained eigenvalues (i.e., proportions relative to the explained variance) showed that the first axis alone explains 24.4% variance, while both axes together explain 28.7%. Of the explanatory variables, a permutation test determined that substrate class (*p* = 0.001, *df* = 3, *F* = 6.712) significantly influenced the variation in epifaunal density.

Morphotaxa with a goodness of fit greater than 0.65 included green sea urchins (*Strongylocentrotus droebachiensis*), slime tube worms (*Myxicola infundibulum*), as well as two indicator taxa including scarlet sea cucumbers (*Psolus fabricii*), and brittle stars (Ophiuroidea spp.) (Fig. 6). Urchins were positively correlated with heterogeneous habitats, such as gravel mix with algal turf. In contrast, brittle stars were positively correlated with homogeneous habitats dominated by fine sediments at higher latitudes as well as diatomaceous sediments. Sites associated with the brittle star-dominated assemblage were mostly found to be correlated with depth, with shallower sites found mostly in diatomaceous sediments. Sites that were positively correlated with fine sediments with pebbles were associated with a high abundance of scarlet sea cucumbers found attached to the larger pebbles and rocks. Slime tube worms, which were exposed above the fine sediments, were also associated with these sites (Fig. 6).

### Charr hotspots & substrate types

**Fig. 7:**
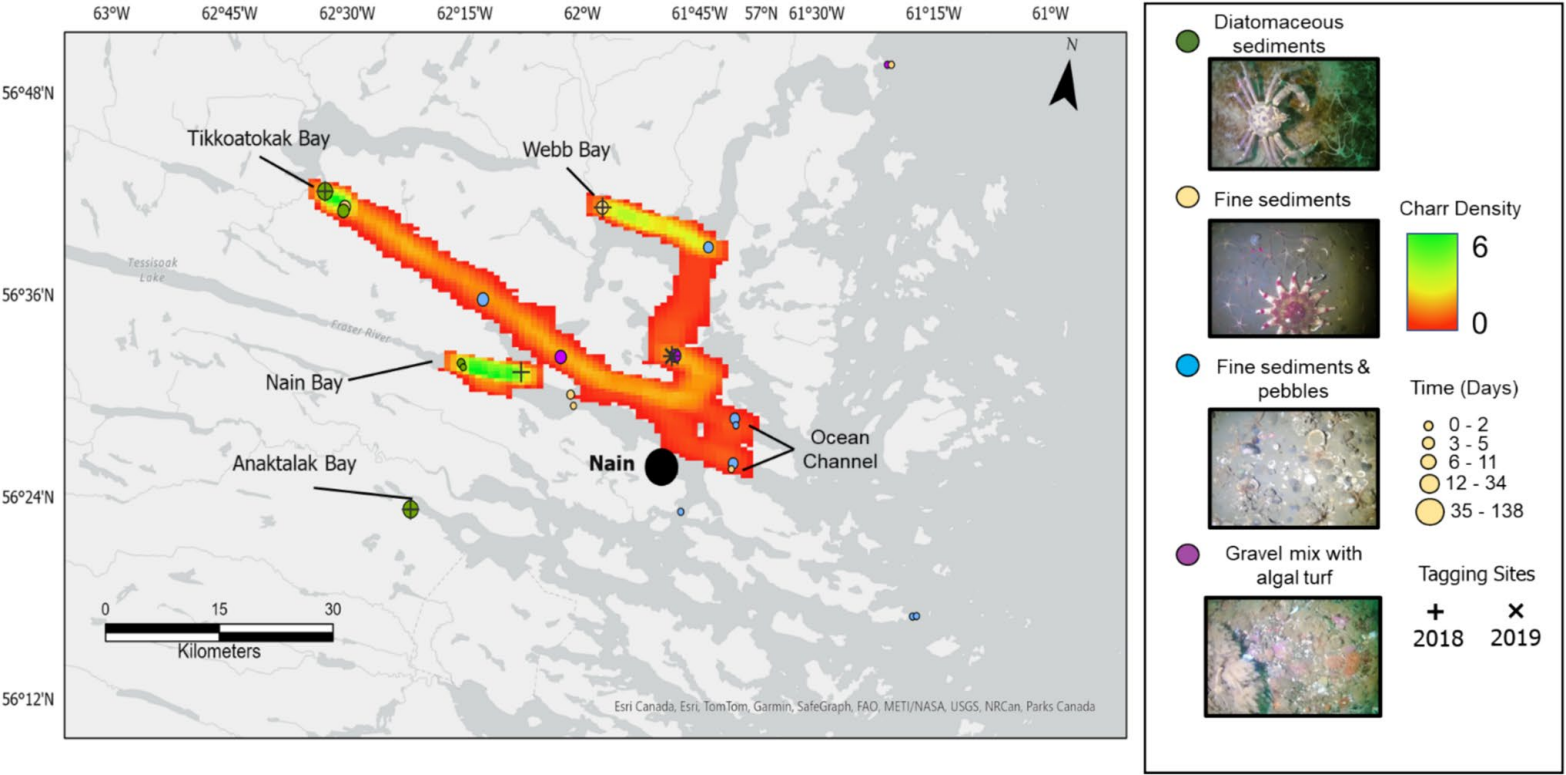
Arctic charr rasterized movement paths (5 × 5 grid) between acoustic receivers and substrate classes. Charr hotspots around Nain are highlighted in green and yellow. Density calculations are based on the number of times an estimated track overlaps with each grid cell (815 m × 387 m). Receivers are coloured by dominant substrate class identified during video annotation

Of the 47 fish in the raw dataset, three were not detected by the same receiver at different times and were filtered out of the analysis leaving a total of 44 animals. Charr hotspots around Nain showed that for the majority of this study’s duration, charr frequented estuarine habitats during their period of marine residency (Fig. 7). Three movement hotspots were identified in Tikkoatokak Bay, Nain Bay, and Webb Bay. Additionally, a high residency area was observed in Anaktalak Bay, where charr remained resident, and thus, this area was not displayed in the movement raster. Migration route coverage for Anaktalak Bay was limited to one receiver; therefore, charr may have used other habitats in proximity which could not be captured. The estuarine habitats were mostly dominated by fine sediment with overlying diatom mats (Table 1). Charr spent 61% of their time near acoustic receivers positioned in diatomaceous substrates. Acoustic receivers located on fine sediment seafloors were used the second most at 16%. Sites with fine sediments covered in pebbles as well as gravel mix with algal turfs were not as frequently occupied by charr, at only 11% and 12%, respectively.

**Table 1:** Cumulative residency time (days) and proportion (%) spent at receivers positioned in four substrate classes

	Fine sediments	Diatomaceous sediments	Fine sediments with pebbles	Gravel mix with algal turf
Cumulative time (days)	70	265	49	52
Proportion (%)	16.0	60.8	11.3	11. 9
Habitat availability (%)	27.0	23.0	36.0	14.0

Proportion of habitat availability (%) also present based on number of acoustic receivers in each substrate class

### Habitat associations & movement

**Fig. 8:**
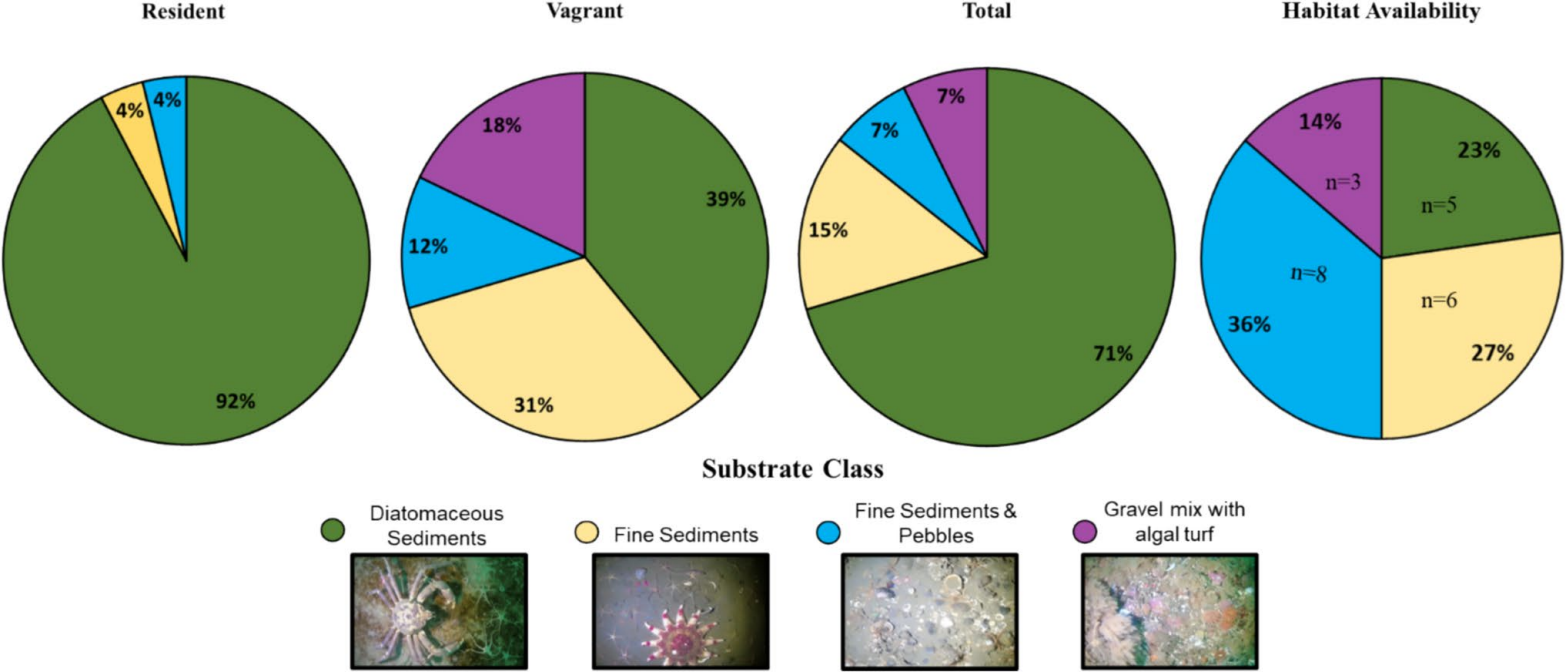
Mean resident, vagrant, and total charr variability per sOfubstrate classification (% use) based on number of detections. Number of individuals (*n*) per substrate class is also indicated. Proportion of habitat availability based on the number of acoustic receivers in each substrate class (*n*) is also shown

Habitat associations were assessed for all charr detected in the study (*n* = 44). Some charr were detected within all substrate classes identified; however, individual variability in substrate use was observed (Online Resource 2, 3). Resident charr represented 59% of the individuals in the study and predominantly remained within estuarine habitats, with the exception of one individual (ID: 2459) who remained in a fjord in proximity to an estuary for the duration of its receiver detections. the resident individuals, 23 were detected only on diatomaceous sediments while the other 2 were detected on fine sediments and fine sediments with pebbles, respectively (Online Resource 2). Vagrant charr tended to frequent harder substrates, like fine sediments with pebbles and gravel mix with algal turf more often; however, on average vagrant charr were also found to frequent diatomaceous sediments and fine sediments the most (Fig. 8). Overall, tagged charr in this study were detected 71% of the time in diatomaceous sediments found in estuaries. The mean weights and lengths of resident charr were not significantly different from the vagrant charr according to the two-sample *t*-test.

### Habitat availability & habitat use

**Fig. 9:**
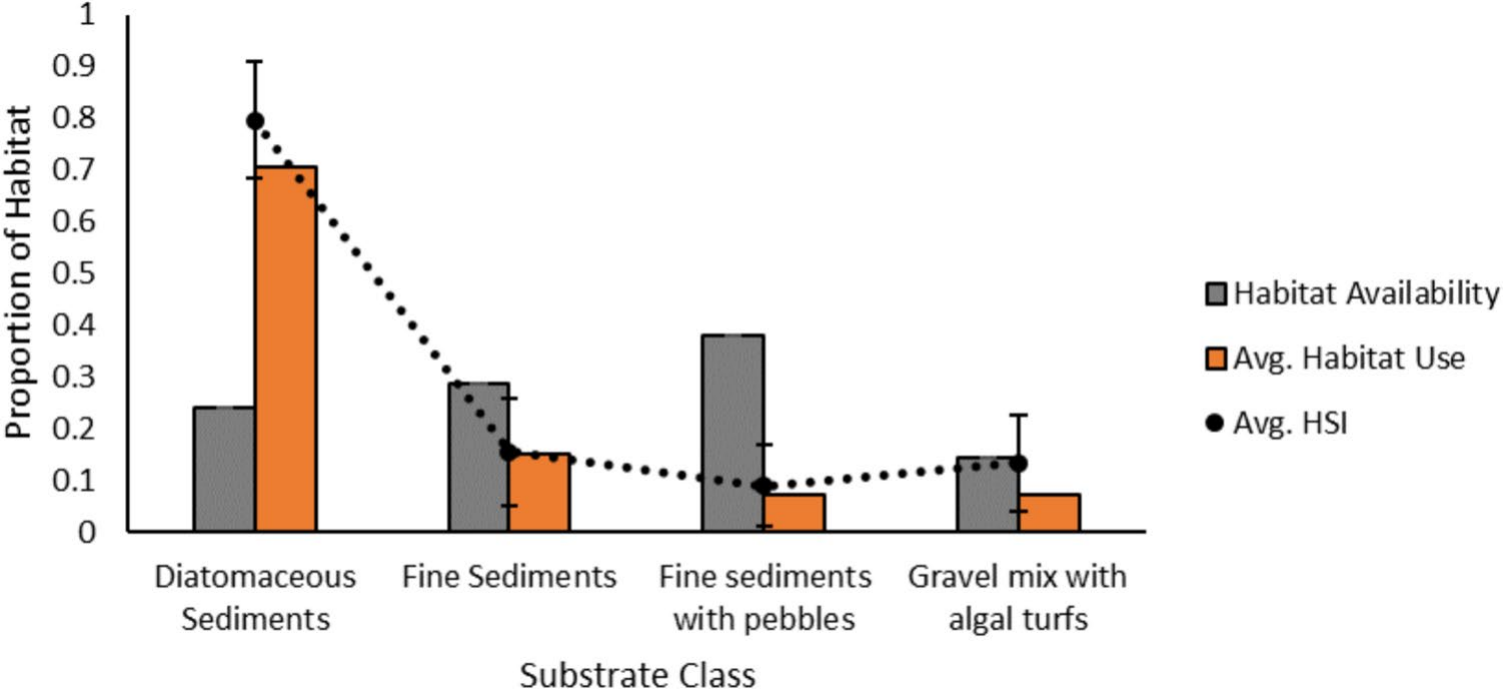
Habitat suitability indices (HSI) for Arctic charr (*n* = 44) by substrate class based on telemetry detections from August 03, 2018 to September 10, 2019. Points indicate the average HSI value for each substrate for all charr. Error bars represent the 95% confidence intervals of the HSI values. Habitat availability was calculated using the number of receivers in each dominant substrate class. Habitat use proportions were based on the telemetry detections of individual charr

On average, the most used substrate class by charr was diatomaceous sediments followed by fine sediments, gravel mix with algal turfs, and fine sediments with pebbles. The mean HSI values exhibited the same pattern (Fig. 9). The substrate class with the most availability in the survey area was fine sediments with pebbles, followed by fine sediments, diatomaceous sediments, and gravel mix with algal turfs. There was a statistically significant difference in the mean habitat used (observed) and habitat available (expected) in the chi squared test (*p* < 0.001, *df* = 3, *χ*^2^ = 58.270).

## Discussion

The spatial representation of epifaunal assemblages in community-identified iKaluk fishing locations and telemetry information from the summer of 2018 and 2019 provides valuable information on charr habitat associations in the marine environment. The findings elaborate on the significance of estuarine habitats, identifying those dominated by fine sediment covered with diatom mats, as charr hotspots during their period of marine residency. Habitat associations of iKaluk are important to establish to address critical knowledge gaps surrounding the ecology of this iconic fish, and aid in the development of targeted conservation and management strategies. While the primary objective was to unveil Arctic charr habitat associations, the video surveys concurrently addressed the scarcity of baseline ecological data in the region. Despite ongoing efforts to expand the protection of northern habitats, potential gaps in critical habitat information persist.

### Charr habitat associations

The utilization of non-estuarine habitats by Arctic charr in Nain exhibits variability year to year (Côté et al. 2021) and has been linked to biological characteristics (e.g., size, sex), local environmental conditions, foraging opportunities, and the proximity to other river systems (Dempson and Kristofferson 1987). While the preference for estuarine habitats during charr marine residency has been well-documented (Spares et al. 2015; Moore et al. 2016; Côté et al. 2021), this study provides new insights into the specific habitat characteristics of these areas. By assessing the habitats associated with high charr usage, we have identified the prevalence of diatomaceous sediments in shallow estuarine environments as a key factor contributing to their residency. This research not only confirms the habitat selection for estuaries but also highlights the potential links of substrate type and productivity patterns within these habitats. Previous studies have proposed that intermediate salinities, optimal temperatures (Harris et al. 2020), and rich foraging opportunities (Miller and Sadro 2003; Moore et al. 2016) drive Arctic charr’s preference for estuaries. In addition to these factors, our research suggests that high productivity in diatom-rich estuaries may also play a crucial role in sustaining Arctic charr populations.

Given the high abundance of benthic diatom mats in Nain’s estuaries, we hypothesize that these areas enhance productivity, supporting the high residency of Arctic charr. Diatom mats are key contributors to coastal ecosystem productivity in the Arctic, influencing the carbon cycle and serving as a foundational food source within estuarine food webs (Glud et al. 2002; Glud et al. 2009; Virta et al. 2020; Liu et al. 2022). In Arctic fjords, Glud et al. (2002) found that benthic diatom primary production is comparable to that of benthic macroalgae and significantly higher than pelagic photosynthesis at depths less than 30 m. The presence of diatom mats in Nain’s estuaries likely boosts populations of local prey species, such as calanoid and cyclopoid copepods (Brown et al. 2012a), which rely on diatoms for nutrition and recruitment success (Irigoien et al. 2000; Michels and Gorb 2015; Hong and Tew 2023). These zooplankton are vital food sources for capelin and sand lance, key prey for Arctic charr (Irigoien et al. 2000; Michels and Gorb 2015). Thus, the productivity patterns driven by benthic primary production suggest that charr may choose to forage in these diatom-rich habitats due to increased prey availability.

Previous diet analyses in Nain have shown pronounced piscivory in nearshore areas compared to coastal headlands, suggesting that estuaries may limit the exploration of non-estuarine habitats when prey is abundant (Côté et al. 2021). A key finding of this study was that both resident and vagrant charr predominantly utilize shallow estuarine habitats with diatomaceous sediments, providing detailed habitat characteristics that were previously unknown. Given that capelin populations have been recovering since their lows in the 1990s (Côté et al. 2021), their abundance in Nain estuaries during this study period may have influenced charr behavior. While diatom mats in these estuaries likely support the abundance of prey, specific observations of these taxa in the video footage were not captured. This absence may be attributed to temporal factors, as capelin may not have been present in the study area during our survey period, and their surface-dwelling behavior, which could limit their visibility to drop cameras positioned near the seabed. However, alternative prey such as sculpin and blennies have been documented in Arctic charr diets (Dempson et al. 2002; Spares et al. 2012), and were dominant species observed in our video surveys. This suggests that these charr may still favor estuarine habitats enriched with diatom mats for foraging.

While the extensive coverage of diatom mats may indicate habitat suitability for charr, this does not imply a direct correlation. However, the presence of diatom mats suggests favorable conditions for supporting a rich prey base, contributing to the overall productivity of these estuarine systems. Chlorophyll *a* concentrations (Chl *a*), serving as a proxy for phytoplankton biomass (Deng et al. 2019), have been correlated with charr distributions and proposed as indicators of food availability (Finstad and Hein 2012; Harris et al. 2020). We hypothesize that high Chl *a* levels in Nain’s estuaries, linked to the extensive benthic diatom mats (Glud et al. 2002, 2009), contribute to the charr hotspots observed in this region.

### Epibenthic community & iKaluk relationships

The benthic community analysis revealed five distinct faunal assemblages associated with different substrate classes. Assemblage 1 was characterized by dense populations of brittle stars (Ophiuroidea spp.) associated with fine and diatomaceous sediments. This ophiuroid-dominated biotope, observed previously in Nain and northern Labrador, plays a key role in energy cycling and substrate stabilization (Geraldi et al. 2017; Rangeley et al. 2022). Similar benthic diatom mats covering fine sediments, along with brittle star dominance, were also documented during drop camera surveys in Young Sound, Greenland fjords (Glud et al. 2002). Two potential iKaluk prey taxa—sculpin (*Cottidae* spp.) and blennies (*Lumpenus* spp.)—were identified in this assemblage (Magnan et al. 2002; Côté et al. 2021). Assemblages 2 and 3, characterized by mixed hard substrates (pebbles, cobbles) and high abundances of sea cucumbers (*Cucumaria frondosa*, *Psolus* spp.) and anemones (*Actiniaria* spp.), were used by vagrant charr, likely serving as feeding and transit habitats (Rikardsen and Amundsen 2005; Côté et al. 2021). Vagrant charr may have also been transiting through these heterogeneous habitats to other estuarine habitats in the region, a behavior consistent with patterns observed in other Arctic regions, such as those studied by Moore et al. (2016) on Victoria Island, Nunavut.

Assemblage 4, found in a less dense charr hotspot in Webb Bay, consisted mainly of fine sediments with sparse fauna. Recent findings indicate the presence of subsea permafrost in the region, which may limit faunal distribution by restricting oxygen and nutrient availability (Vonk et al. 2015; Normandeau et al. 2024a). The sampling effort for this assemblage was limited, as indicated by the species accumulation curve; however, further drop-camera images collected from this area in 2022 and 2023 identified the same patterns of fine sediments with sparse fauna (Limoges et al. 2023; Normandeau et al. 2024b). The final assemblage, found in a charr hotspot in Anaktalak Bay, was characterized by tube-dwelling anemones (*Ceriantharia* spp.) over a diatom-covered seafloor. While this area is home to a nickel–copper–cobalt mine, the implications of mining on local ecosystems remain poorly understood. The notable scarcity of brittle stars in this assemblage may relate to effluent contamination patterns (de Moura Barboza et al. 2015), while the presence of diatom mats could indicate sedimentation related to environmental disturbances (Kahlmeyer 2009).

While this study provides valuable insights into the habitat associations of Arctic charr in Nain, several caveats warrant consideration. First, the telemetry data underscore the importance of estuary use, a finding supported by local knowledge and aligned with published studies from other regions (Miller and Sadro 2003; Moore et al. 2016; Harris et al. 2020). The existing state of knowledge is sufficiently robust that habitat associations could have been assessed without telemetry data; however, these data confirm the high usage of estuaries in our study period. Calculating habitat use in the HSI presents limitations, as detection numbers were used to assess habitat utilization, leading to duplicate detections due to overlapping receiver ranges. These duplicates account for a minor percentage (5%) of overall detections, and our detection event methodology—examining residency time and accounting for these overlaps—supports the main trend favoring diatomaceous sediment habitats. Additionally, potential habitat-specific differences in acoustic receiver detection range may exist; however, these differences would likely reinforce the observed results, as shallow estuarine waters are less acoustically favorable than deeper, less stratified areas.

The receiver locations selected may not fully represent the habitat diversity used by charr. Although habitats near receivers tended to be uniform, further insights could be gained through finer-scale data collection, such as a positioning telemetry system. Furthermore, while the pronounced use of estuaries by vagrant fish alleviates concerns about the influence of release locations, one of two fish released at fjord sites also highly utilized estuaries, emphasizing their significance. Finally, the coverage of acoustic receivers in Anaktalak Bay posed challenges for capturing charr movements, as we lacked receivers outside the estuary. Despite this limitation, Anaktalak Bay is recognized as an important fishing location for the local community, which justifies its inclusion in the analysis. These caveats highlight the complexities of studying habitat use in dynamic environments and stress the importance of interpreting results within this broader context.

### Environmental change & management implications

The diatom mats observed throughout charr hotspots in this study likely serve as a critical food source for benthic organisms, including copepods and other zooplankton, which are vital in the marine food web. As warming continues across Nunatsiavut, an earlier spring bloom is expected to alter sympagic, benthic, and pelagic productivity, affecting the diet and habitat use of Arctic charr (Falardeau et al. 2022). Earlier sea ice breakup could shift bloom dynamics, providing substantial biomass for benthic organisms but also impacting prey availability, which may disrupt the charr trophic web (Boetius et al. 2013; AMAP 2017). Similar effects have been observed in polar cod (*Boreogadus saida*) in the Canadian Arctic (McNicholl et al. 2016). These changes may force charr to adapt their migration, feeding behaviors, and energy expenditure for survival (McNicholl et al. 2016; Secretariat 2017).

Our research identified diatomaceous sediments around Arctic charr hotspots in estuarine habitats, which are key areas for charr during their marine residency. The presence of these sediments suggests high productivity in these estuaries, likely supporting important Arctic charr prey and further reinforcing the importance of these regions for Arctic charr. Although this study was limited by the number and distribution of receivers, the results suggest that estuarine habitats, particularly those characterized by diatomaceous sediments, are critical for the survival and stability of charr populations. Given that charr in the Nain region are currently experiencing challenges including a decline in active fishers and inadequate stock assessments (Kourantidou et al. 2024), it is essential to integrate management strategies that balance cultural activities, like fishing, with conservation and economic development. Improving stock assessment frequency and accuracy, alongside habitat protection efforts, will increase the sustainability of Arctic charr stocks and support the communities that depend on them throughout the ongoing environmental changes. The insights gained from this study aim to support the Imappivut initiative and guide the sustainable management of Nain’s iKaluk fishery, ultimately preserving this essential resource and its ecosystem for Nunatsiavut’s Inuit communities in the face of climate change.

## Supplementary Information

Below is the link to the electronic supplementary material.

Supplementary file1 (DOCX 952 KB)

## Data Availability

The datasets generated during and/or analyzed during the current study are available from the corresponding author on reasonable request and will soon be publicly accessible on the Federated Research Data Repository.
