# Supplementary material for: Associations between iKaluk/Arctic charr (Salvelinus alpinus) and estuarine benthic diatom habitats in nearshore Nunatsiavut waters
Source: Polar Biol. 2025 Jan 2;48(1):20. doi: 10.1007/s00300-024-03323-z (PMC11693620; doi:10.1007/s00300-024-03323-z)
Supplement: Supplementary file 1 — Supplementary file1 (DOCX 952 KB) [file 300_2024_3323_MOESM1_ESM.docx]

**Supplementary Material**

**Online Resource 1** Full set of environmental variables per site. List includes cluster, visual footprint area ($m^{2}$), transect length (m), depth (m), distance to freshwater (m), substrate classification (subclass), habitat type, mean latitude (Lat), mean longitude (Lon), and percentage (%) of substrates: fine sediments, gravel, pebbles, cobbles, boulders, diatoms, coralline algae, red seaweeds (Porphyra spp.) and brown seaweeds (Laminaria spp.) (Algae), and shellhash

| **Site** | **Cluster** | **Area** | **Length** | **Depth** | **Dist2fresh** | **Habitat** | **Lat** | **Lon** | **Subclass** | **Fine sediments** | **Gravel** | **Pebbles** | **Cobbles** | **Boulders** | **Bedrock** | **Diatoms** | **Coralline algae** | **Algae** | **Shellhash** | **Unidentified** |
| --- | --- | --- | --- | --- | --- | --- | --- | --- | --- | --- | --- | --- | --- | --- | --- | --- | --- | --- | --- | --- |
| 1 | 1 | 19 | 41 | 226 | 20337 | Estuary | 56.592 | -61.906 | FS | 94 | 0 | 0 | 0 | 0 | 0 | 0 | 0 | 0 | 0 | 6 |
| 2 | 1 | 16 | 25 | 260 | 19696 | Estuary | 56.603 | -61.915 | FS | 97 | 0 | 0 | 0 | 0 | 0 | 0 | 0 | 0 | 0 | 3 |
| 3 | 1 | 10 | 27 | 33 | 6012 | Estuary | 56.605 | -62.138 | DS | 22 | 0 | 0 | 0 | 0 | 0 | 75 | 0 | 3 | 0 | 0 |
| 4 | 1 | 10 | 24 | 53 | 4937 | Estuary | 56.614 | -62.155 | DS | 20 | 0 | 0 | 0 | 0 | 0 | 79 | 0 | 1 | 0 | 0 |
| 5 | 1 | 7 | 34 | 47 | 5281 | Estuary | 56.610 | -62.150 | DS | 13 | 0 | 0 | 0 | 0 | 0 | 85 | 0 | 1 | 0 | 1 |
| 6 | 2 | 16 | 60 | 169 | 26678 | Fjord | 56.639 | -61.947 | GMA | 0 | 53 | 20 | 1 | 3 | 10 | 0 | 5 | 7 | 0 | 1 |
| 7 | 1 | 20 | 17 | 340 | 24942 | Fjord | 56.681 | -62.128 | FSP | 87 | 0 | 7 | 0 | 0 | 0 | 0 | 0 | 0 | 0 | 5 |
| 8 | 1 | 14 | 53 | 131 | 3878 | Estuary | 56.746 | -62.448 | FS | 99 | 0 | 0 | 0 | 0 | 0 | 0 | 0 | 0 | 0 | 1 |
| 9 | 1 | 12 | 49 | 76 | 4042 | Estuary | 56.742 | -62.449 | DS | 32 | 0 | 0 | 0 | 0 | 0 | 67 | 0 | 0 | 0 | 1 |
| 10 | 1 | 21 | 55 | 69 | 713 | Estuary | 56.758 | -62.495 | DS | 44 | 0 | 0 | 0 | 0 | 0 | 55 | 0 | 1 | 0 | 0 |
| 11 | 3 | 11 | 31 | 118 | 40111 | Fjord | 56.609 | -61.561 | FSP | 86 | 0 | 2 | 1 | 0 | 0 | 2 | 0 | 1 | 0 | 8 |
| 12 | 1 | 13 | 35 | 275 | 40490 | Fjord | 56.603 | -61.557 | FSP | 92 | 0 | 5 | 1 | 0 | 0 | 0 | 0 | 1 | 0 | 1 |
| 13 | 1 | 14 | 33 | 307 | 38397 | Fjord | 56.565 | -61.552 | FSP | 64 | 0 | 15 | 2 | 1 | 0 | 0 | 0 | 0 | 17 | 1 |
| 14 | 1 | 11 | 23 | 277 | 38950 | Fjord | 56.559 | -61.555 | FS | 99 | 0 | 0 | 0 | 0 | 0 | 0 | 0 | 1 | 0 | 0 |
| 15 | 3 | 27 | 66 | 236 | 28843 | Fjord | 56.507 | -61.649 | FSP | 41 | 6 | 17 | 1 | 3 | 0 | 0 | 0 | 1 | 29 | 2 |
| 16 | 3 | 25 | 72 | 80 | 36500 | Fjord | 56.658 | -61.530 | GMA | 23 | 32 | 3 | 1 | 0 | 0 | 0 | 8 | 24 | 0 | 9 |
| 17 | 2 | 17 | 61 | 119 | 25433 | Fjord | 56.661 | -61.705 | GMA | 0 | 51 | 9 | 1 | 3 | 1 | 0 | 1 | 20 | 11 | 3 |
| 18 | 3 | 10 | 65 | 95 | 15946 | Fjord | 56.775 | -61.666 | FSP | 84 | 0 | 2 | 1 | 1 | 0 | 0 | 1 | 6 | 2 | 2 |
| 19 | 4 | 10 | 86 | 56 | 1221 | Estuary | 56.795 | -61.902 | FS | 97 | 0 | 0 | 0 | 0 | 0 | 1 | 0 | 1 | 0 | 0 |
| 20 | 5 | 23 | 37 | 70 | 301 | Estuary | 56.459 | -62.218 | DS | 52 | 0 | 0 | 0 | 0 | 0 | 41 | 0 | 5 | 0 | 1 |
| 21 | 1 | 33 | 54 | 161 | 3679 | Estuary | 56.496 | -62.049 | FS | 99 | 0 | 0 | 0 | 0 | 0 | 0 | 0 | 0 | 0 | 0 |
| 22 | 3 | 48 | 93 | 118 | 41487 | Estuary | 56.989 | -61.336 | GMA | 77 | 11 | 5 | 0 | 0 | 0 | 0 | 5 | 0 | 0 | 0 |
| 23 | 1 | 34 | 83 | 155 | 41949 | Estuary | 56.990 | -61.328 | FS | 100 | 0 | 0 | 0 | 0 | 0 | 0 | 0 | 0 | 0 | 0 |
| 24  25 | 3  3 | 72  66 | 187  172 | 148  121 | 58971  58460 | Coastal  Coastal | 56.445  56.444 | -61.125  -61.132 | FSP  FSP | 70  66 | 0  1 | 29  22 | 0  1 | 1  1 | 0  0 | 0  0 | 0  9 | 0  0 | 0  0 | 0  0 |

**Online Resource 2** Metadata associated with telemetered charr. Includes length (cm), weight (g), release date, date and time of first and last detection, number of days detected, number of days passed since their release, number of receivers, number of detections, and acoustic receiver IDs

| **Fish** | **Length.cm** | **Weight.g** | **Release date** | **Date & time (UTC) of first detection** | **Date & time (UTC) of last detection** | **# Days detected** | **# Days since release** | **# Unique receiver** | **Detection Number** | **Status** | **Acoustic Receiver** | **Substrate** | **Site** | **Habitat** |
| --- | --- | --- | --- | --- | --- | --- | --- | --- | --- | --- | --- | --- | --- | --- |
| 2438 | 60.5 | 3855.54 | 02-Aug-18 | 2018-08-03 12:10 | 2018-08-03 14:13 | 0 | 1 | 1 | 8 | Resident | 124358 | FS | 19 | Estuary |
| 2441 | 48.7 | 1587.57 | 02-Aug-18 | 2018-08-03 2:33 | 2018-08-11 11:09 | 8 | 9 | 1 | 261 | Resident | 124408 | DS | 10 | Estuary |
| 2443 | 51 | 1814.37 | 02-Aug-18 | 2018-08-03 1:54 | 2018-08-03 11:40 | 0 | 1 | 1 | 229 | Resident | 124408 | DS | 10 | Estuary |
| 2445 | 50.8 | 1814.37 | 02-Aug-18 | 2018-08-03 2:08 | 2018-08-19 9:14 | 16 | 17 | 1 | 123 | Resident | 124408 | DS | 10 | Estuary |
| 2447 | 48 | 1814.37 | 02-Aug-18 | 2018-08-03 10:10 | 2018-08-04 9:35 | 1 | 2 | 1 | 17 | Resident | 124408 | DS | 10 | Estuary |
| 2449 | 53 | 2041.17 | 02-Aug-18 | 2018-08-03 1:17 | 2018-08-22 8:16 | 19 | 20 | 1 | 27 | Resident | 124408 | DS | 10 | Estuary |
| 2459 | 38.2 | 635.03 | 31-Jul-19 | 2019-08-10 11:51 | 2019-08-13 16:26 | 3 | 13 | 1 | 7 | Resident | 547227 | FSP | 18 | Fjord |
| 2465 | 45.5 | 1133.98 | 04-Aug-18 | 2018-08-06 19:21 | 2019-07-18 17:27 | 346 | 348 | 1 | 34 | Resident | 547211 | DS | 20 | Estuary |
| 2466 | 49 | 1587.57 | 04-Aug-18 | 2018-08-04 16:02 | 2019-07-14 8:59 | 344 | 344 | 1 | 1980 | Resident | 547211 | DS | 20 | Estuary |
| 2467 | 45 | 1360.78 | 04-Aug-18 | 2018-08-04 18:54 | 2019-07-21 20:56 | 351 | 351 | 1 | 60 | Resident | 547211 | DS | 20 | Estuary |
| 2469 | 48 | 1587.57 | 04-Aug-18 | 2018-08-05 20:24 | 2018-08-13 18:39 | 8 | 9 | 1 | 794 | Resident | 547211 | DS | 20 | Estuary |
| 4364 | 53 | 2041.17 | 04-Aug-18 | 2018-08-04 15:05 | 2018-08-08 20:18 | 4 | 4 | 1 | 344 | Resident | 547211 | DS | 20 | Estuary |
| 4365 | 50 | 1360.78 | 04-Aug-18 | 2018-08-11 5:21 | 2018-08-11 5:29 | 0 | 7 | 1 | 5 | Resident | 547211 | DS | 20 | Estuary |
| 4366 | 47.1 | 1360.78 | 04-Aug-18 | 2018-08-05 7:07 | 2019-07-16 6:58 | 345 | 346 | 1 | 91 | Resident | 547211 | DS | 20 | Estuary |
| 4367 | 49.5 | 1587.57 | 04-Aug-18 | 2019-06-02 15:37 | 2019-07-23 8:06 | 51 | 353 | 1 | 9 | Resident | 547211 | DS | 20 | Estuary |
| 4368 | 47.5 | 1814.37 | 04-Aug-18 | 2019-06-03 5:24 | 2019-07-10 6:35 | 37 | 340 | 1 | 1260 | Resident | 547211 | DS | 20 | Estuary |
| 4369 | 41.5 | 1814.37 | 04-Aug-18 | 2018-08-04 14:08 | 2018-08-09 0:27 | 5 | 5 | 1 | 374 | Resident | 547211 | DS | 20 | Estuary |
| 4371 | 48.2 | 1133.98 | 04-Aug-18 | 2018-08-05 12:35 | 2019-06-25 14:53 | 324 | 325 | 1 | 972 | Resident | 547211 | DS | 20 | Estuary |
| 4372 | 45 | 1587.57 | 04-Aug-18 | 2018-08-04 20:09 | 2019-07-24 15:25 | 354 | 354 | 1 | 249 | Resident | 547211 | DS | 20 | Estuary |
| 4373 | 46 | 1360.78 | 04-Aug-18 | 2018-08-11 18:21 | 2018-08-11 18:32 | 0 | 7 | 1 | 2 | Resident | 547211 | DS | 20 | Estuary |
| 4375 | 48.3 | 1587.57 | 04-Aug-18 | 2018-08-04 18:13 | 2019-07-07 2:09 | 337 | 337 | 1 | 1387 | Resident | 547211 | DS | 20 | Estuary |
| 4378 | 51.5 | 1814.37 | 03-Aug-18 | 2018-08-03 15:33 | 2018-08-03 15:39 | 0 | 0 | 1 | 5 | Resident | 122386 | DS | 4 | Estuary |
| 4383 | 58.5 | 2721.55 | 02-Aug-18 | 2018-08-03 4:26 | 2018-09-03 6:31 | 31 | 32 | 1 | 14 | Resident | 124408 | DS | 10 | Estuary |
| 4386 | 52 | 1814.37 | 02-Aug-18 | 2018-08-03 12:32 | 2018-08-03 12:52 | 0 | 1 | 1 | 5 | Resident | 124408 | DS | 10 | Estuary |
| 4388 | 63.5 | 3401.94 | 02-Aug-18 | 2018-08-03 7:00 | 2018-08-03 13:03 | 0 | 1 | 1 | 108 | Resident | 124408 | DS | 10 | Estuary |
| 2436 | 47 | 1814.37 | 31-Jul-18 | 2018-08-03 9:15 | 2019-07-24 21:27 | 355 | 358 | 2 | 128 | Vagrant | 547227, 124358 | FS, FSP | 18, 19 | Fjord, Estuary |
| 2437 | 58 | 3175.15 | 02-Aug-18 | 2018-08-03 9:11 | 2019-07-21 0:13 | 352 | 353 | 2 | 168 | Vagrant | 547227, 124358 | FS, FSP | 18, 19 | Fjord, Estuary |
|  |  |  |  |  |  |  |  |  |  |  |  |  |  |  |
| 2439 | 52 | 1814.37 | 02-Aug-18 | 2018-08-03 11:09 | 2019-09-03 10:24 | 396 | 397 | 8 | 606 | Vagrant | 547207, 547209, 547210, 547215, 547216, 547225, 547228, 124408 | FSP, FS, FS, FSP, DS, GMA, FSP, DS | 12, 14, 8, 11, 9, 6, 13, 10 | Fjord, Fjord, Estuary, Fjord, Estuary, Fjord, Fjord, Estuary |
| 2440 | 53 | 1814.37 | 02-Aug-18 | 2018-08-03 0:00 | 2019-07-31 18:03 | 362 | 363 | 6 | 3211 | Vagrant | 547210, 547213, 547216, 547225, 547229, 124408 | FS, FSP, DS, GMA, FSP, DS | 8, 17, 9, 6, 7, 10 | Estuary, Fjord, Estuary, Fjord, Fjord, Estuary |
| 2444 | 46.5 | 1360.78 | 02-Aug-18 | 2018-08-03 3:30 | 2019-08-07 21:09 | 369 | 370 | 8 | 4568 | Vagrant | 547201, 547210, 547216, 547221, 547225, 547229, 122386, 124408 | FS, FS, DS, FS, GMA, FSP, DS, DS | 2, 8, 9, 1, 6, 7, 4, 10 | Estuary, Estuary, Estuary, Estuary, Fjord, Fjord, Estuary, Estuary |
| 2446 | 49.5 | 1587.57 | 02-Aug-18 | 2018-08-03 1:12 | 2018-08-04 15:04 | 1 | 2 | 3 | 402 | Vagrant | 547210, 547216, 124408 | FS, DS, DS | 8, 9, 10 | Estuary, Estuary, Estuary |
| 2455 | 34.5 | NA | 01-Aug-19 | 2019-08-04 15:23 | 2019-08-23 23:30 | 19 | 22 | 4 | 258 | Vagrant | 547210, 547216, 547229, 124408 | FS, DS, FSP, DS | 8, 9, 7, 10 | Estuary, Estuary, Fjord, Estuary |
|  |  |  |  |  |  |  |  |  |  |  |  |  |  |  |
| 2456 | 41.7 | NA | 01-Aug-19 | 2019-08-02 17:20 | 2019-08-03 19:26 | 1 | 2 | 4 | 78 | Vagrant | 547210, 547216, 547229, 124408 | FS, DS, FSP, DS | 8, 9, 7, 10 | Estuary, Estuary, Fjord, Estuary |
| 2460 | 42.7 | 512.56 | 31-Jul-19 | 2019-08-01 11:25 | 2019-08-08 9:36 | 7 | 8 | 6 | 210 | Vagrant | 547210, 547213, 547216, 547225, 547229, 124408 | FS, GMA, DS, GMA, FSP, DS | 8, 17, 9, 6, 7, 10 | Estuary, Fjord, Estuary, Fjord, Fjord, Estuary |
| 2461 | 49.5 | 952.54 | 31-Jul-19 | 2019-08-08 18:18 | 2019-08-09 18:34 | 1 | 9 | 2 | 17 | Vagrant | 547213, 547221 | GMA, FS | 17, 1 | Fjord, Estuary |
| 2462 | 43.5 | 952.54 | 31-Jul-19 | 2019-08-05 21:25 | 2019-08-24 8:49 | 19 | 24 | 5 | 150 | Vagrant | 547201, 547213, 547225, 122386, 124881 | FS, GMA, GMA, DS, DS | 2, 17, 6, 4, 5 | Estuary, Fjord, Fjord, Estuary, Estuary |
| 2463 | 47 | 961.62 | 31-Jul-19 | 2019-08-09 15:45 | 2019-08-18 14:43 | 9 | 18 | 2 | 83 | Vagrant | 547213, 547227 | GMA, FSP | 17, 18 | Fjord, Fjord |
| 2468 | NA | 680.39 | 31-Jul-19 | 2019-08-01 5:32 | 2019-08-12 22:18 | 11 | 12 | 6 | 114 | Vagrant | 547210, 547213, 547216, 547225, 547229, 124408 | FS, GMA, DS, GMA, FSP, DS | 8, 17, 9, 6, 7, 10 | Estuary, Fjord, Estuary, Fjord, Fjord, Estuary |
| 4376 | 50 | 1133.98 | 02-Aug-18 | 2018-08-03 5:46 | 2018-08-04 11:20 | 1 | 2 | 3 | 157 | Vagrant | 547210, 547216, 124408 | FS, DS, DS | 8, 9, 10 | Estuary, Estuary, Estuary |
| 4377 | 58.5 | 2041.17 | 02-Aug-18 | 2018-08-03 2:17 | 2018-08-04 12:53 | 1 | 2 | 3 | 330 | Vagrant | 547210, 547216, 124408 | FS, DS, DS | 8, 9, 10 | Estuary, Estuary, Estuary |
| 4380 | 49.5 | 1360.78 | 03-Aug-18 | 2019-06-06 14:41 | 2019-06-12 12:37 | 6 | 313 | 4 | 76 | Vagrant | 547212, 547221, 124881, 122386 | FSP, FS, DS, DS | 15, 1, 5, 4 | Estuary, Estuary, Estuary, Estuary |
| 4381 | 44.5 | 453.59 | 03-Aug-18 | 2018-08-04 15:28 | 2018-08-04 15:49 | 0 | 1 | 2 | 8 | Vagrant | 122386, 124881 | DS, DS | 4, 5 | Estuary, Estuary |
| 4382 | 43 | 2041.17 | 02-Aug-18 | 2018-08-03 2:33 | 2018-08-04 14:24 | 1 | 2 | 3 | 451 | Vagrant | 547210, 547216, 124408 | FS, DS, DS | 8, 9, 10 | Estuary, Estuary, Estuary |
| 4384 | 57.5 | 2267.96 | 02-Aug-18 | 2018-08-03 9:09 | 2018-09-10 10:08 | 38 | 39 | 3 | 778 | Vagrant | 547210, 547216, 124408 | FS, DS, DS | 8, 9, 10 | Estuary, Estuary, Estuary |

**Online Resource 3**  Individual charr variability per substrate classification (% use) based on number of detections

| **Fish ID** | **Diatomaceous Sediments** | **Fine Sediments** | **Fine sediments with pebbles** | **Gravel mix with algal turfs** |
| --- | --- | --- | --- | --- |
| 2436 | 0.0 | 60.2 | 39.8 | 0.0 |
| 2437 | 0.0 | 92.3 | 7.7 | 0.0 |
| 2438 | 0.0 | 100.0 | 0.0 | 0.0 |
| 2439 | 26.6 | 12.7 | 54.5 | 6.3 |
| 2440 | 61.4 | 12.7 | 2.5 | 23.4 |
| 2441 | 100.0 | 0.0 | 0.0 | 0.0 |
| 2443 | 100.0 | 0.0 | 0.0 | 0.0 |
| 2444 | 55.0 | 35.2 | 0.7 | 9.1 |
| 2445 | 100.0 | 0.0 | 0.0 | 0.0 |
| 2446 | 81.8 | 18.2 | 0.0 | 0.0 |
| 2447 | 100.0 | 0.0 | 0.0 | 0.0 |
| 2449 | 100.0 | 0.0 | 0.0 | 0.0 |
| 2455 | 17.8 | 29.5 | 52.7 | 0.0 |
| 2456 | 55.1 | 38.5 | 6.4 | 0.0 |
| 2459 | 0.0 | 0.0 | 100.0 | 0.0 |
| 2460 | 34.8 | 18.6 | 1.4 | 45.2 |
| 2461 | 0.0 | 76.5 | 0.0 | 23.5 |
| 2462 | 9.3 | 10.7 | 0.0 | 80.0 |
| 2463 | 0.0 | 0.0 | 26.5 | 73.5 |
| 2465 | 100.0 | 0.0 | 0.0 | 0.0 |
| 2466 | 100.0 | 0.0 | 0.0 | 0.0 |
| 2467 | 100.0 | 0.0 | 0.0 | 0.0 |
| 2468 | 20.2 | 8.8 | 10.5 | 60.5 |
| 2469 | 100.0 | 0.0 | 0.0 | 0.0 |
| 4364 | 100.0 | 0.0 | 0.0 | 0.0 |
| 4365 | 100.0 | 0.0 | 0.0 | 0.0 |
| 4366 | 100.0 | 0.0 | 0.0 | 0.0 |
| 4367 | 100.0 | 0.0 | 0.0 | 0.0 |
| 4368 | 100.0 | 0.0 | 0.0 | 0.0 |
| **Fish ID** | **Diatomaceous Sediments** | **Fine Sediments** | **Fine sediments with pebbles** | **Gravel mix with algal turfs** |
| 4369 | 100.0 | 0.0 | 0.0 | 0.0 |
| 4371 | 100.0 | 0.0 | 0.0 | 0.0 |
| 4372 | 100.0 | 0.0 | 0.0 | 0.0 |
| 4373 | 100.0 | 0.0 | 0.0 | 0.0 |
| 4375 | 100.0 | 0.0 | 0.0 | 0.0 |
| 4376 | 52.2 | 47.8 | 0.0 | 0.0 |
| 4377 | 63.3 | 36.7 | 0.0 | 0.0 |
| 4378 | 100.0 | 0.0 | 0.0 | 0.0 |
| 4380 | 82.9 | 10.5 | 6.6 | 0.0 |
| 4381 | 100.0 | 0.0 | 0.0 | 0.0 |
| 4382 | 44.1 | 55.9 | 0.0 | 0.0 |
| 4383 | 100.0 | 0.0 | 0.0 | 0.0 |
| 4384 | 97.7 | 2.3 | 0.0 | 0.0 |
| 4386 | 100.0 | 0.0 | 0.0 | 0.0 |
| 4388 | 100.0 | 0.0 | 0.0 | 0.0 |
| avg | 70.5 | 15.2 | 7.0 | 7.3 |


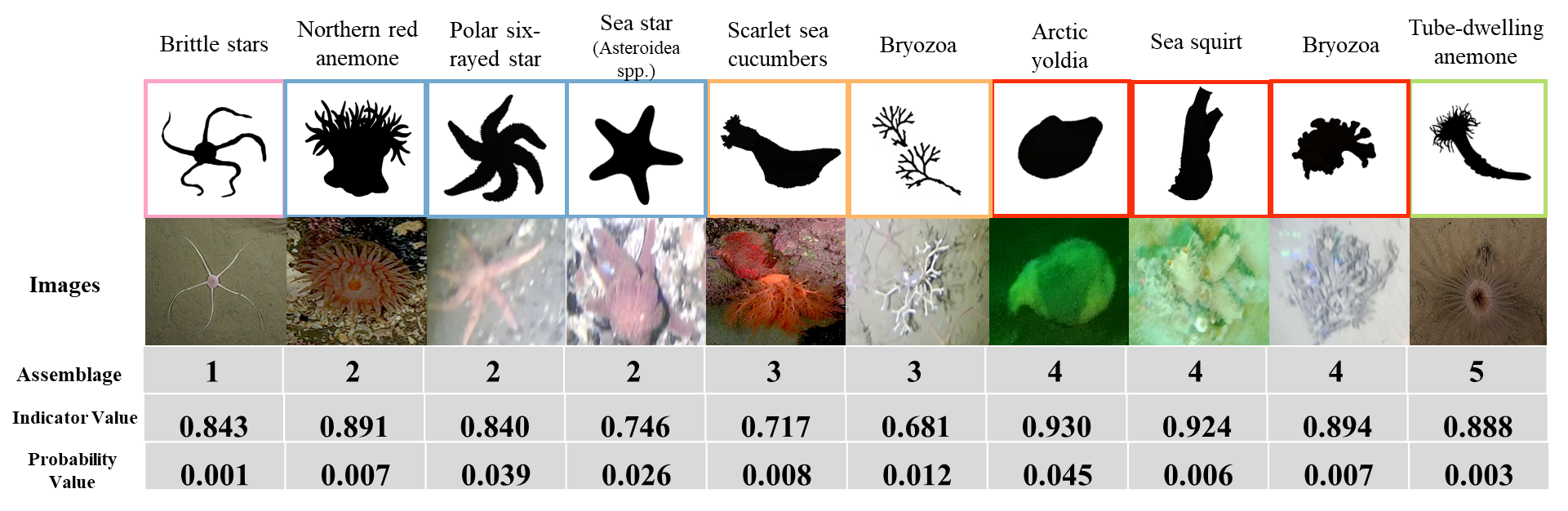


**Online Resource 4** Indicator taxa with associated indicator and probability values for 5 epifaunal assemblages

**Online Resource 5** Total abundance of morphotaxa throughout survey in the Nain region of NL. Status denotes dominant (x) and indicator taxa (*)

| Status | Morphotaxa | Total abundance |
| --- | --- | --- |
|  | Actiniaria spp. | 17 |
|  | Amphipoda spp. | 16 |
| x * | Ascidiacea sp.1 | 1044 |
| x | Ascidiacea sp.2 | 129 |
|  | Asterias forbesi | 26 |
| x | Asteroidea sp.11 | 22 |
|  | Asteroidea spp.003 | 20 |
| x | Boltenia ovifera | 626 |
|  | Bryozoa.sp.003 | 30 |
| x | Bryozoa.sp.004 | 126 |
| x * | Bryozoa.sp.1 | 678 |
| x | Bryozoa.sp.2 | 98 |
| x * | Bryozoa.sp.4 | 74 |
| x | Bryozoa.sp.6 | 384 |
|  | Buccinum spp. | 19 |
| x * | Ceriantharia spp. | 11874 |
|  | Chionoecetes opilio | 78 |
| x | Chlamys islandica | 264 |
| x | Cottidae spp. | 376 |
| x | Crossaster papposus | 367 |
|  | Ctenodiscus crispatus | 10 |
| x | Cucumaria frodosa | 473 |
|  | Halcampa arctica | 56 |
|  | Haliclona sp.2 | 50 |
| x | Halocynthia pyriformis | 109 |
|  | Heliometra glacialis | 242 |
| x | Henricia sanguinolenta | 172 |
| x | Hyas araneus | 146 |
| x | Hyas coarctatus | 77 |
| x * | Leptasterias polaris | 514 |
| Status | Morphotaxa | Total abundance |
| x | Lumpenus lampretaeformis | 360 |
|  | Lycodes vahlii | 88 |
|  | Mya truncata | 32 |
|  | Myoxocephalus scorpius | 58 |
|  | Myoxocephalus spp. | 30 |
| x | Myxicola infundibulum | 1845 |
| x | Nephtheidae sp. 3 | 535 |
|  | Nephtheidae sp.1 | 54 |
| x * | Ophiuroidea sp.1 | 194993 |
| x | Ophiuroidea sp.2 | 5633 |
|  | Pagurus spp. | 31 |
| x | Pandalus spp. | 540 |
| x | Polinices heros | 34 |
|  | Polychaeta spp. | 11 |
|  | Porifera sp.11 | 72 |
| x | Porifera sp.13 | 515 |
|  | Porifera sp.21 | 38 |
| x | Porifera sp.3 | 690 |
|  | Porifera sp.5 | 14 |
|  | Poriferasp 22 | 42 |
| * | Portlandia arctica | 22 |
| x * | Psolus fabricii | 2725 |
| x | Psolus phantapus | 9380 |
| x | Psolus sp.1 | 394 |
|  | Ptychogena lactea | 19 |
|  | Sabellida spp. | 16 |
| x | Sagittidae spp. | 91 |
|  | Scypha spp. | 12 |
| x | Stomphia coccinea | 121 |
| x | Stronglyocentrotus droebachiensis | 6106 |
| x * | Urticina felina | 5412 |
|  | Zoarcidae sp.1 | 5 |
| x | Zoarcidae sp.2 | 21 |
